# Trends in the Usage of Statistical Software and Their Associated Study Designs in Health Sciences Research: A Bibliometric Analysis

**DOI:** 10.7759/cureus.12639

**Published:** 2021-01-11

**Authors:** Emad Masuadi, Mohamud Mohamud, Muhannad Almutairi, Abdulaziz Alsunaidi, Abdulmohsen K Alswayed, Omar F Aldhafeeri

**Affiliations:** 1 Research Unit/Biostatistics, King Saud bin Abdulaziz University for Health Sciences, College of Medicine/King Abdullah International Medical Research Centre, Riyadh, SAU; 2 Research Unit/Epidemiology, King Saud bin Abdulaziz University for Health Sciences, College of Medicine, Riyadh, SAU; 3 Medicine, King Saud bin Abdulaziz University for Health Sciences, College of Medicine, Riyadh, SAU

**Keywords:** statistical software, study design, healthcare publications, spss, stata, sas, pubmed

## Abstract

Background

The development of statistical software in research has transformed the way scientists and researchers conduct their statistical analysis. Despite these advancements, it was not clear which statistical software is mainly used for which research design thereby creating confusion and uncertainty in choosing the right statistical tools. Therefore, this study aimed to review the trend of statistical software usage and their associated study designs in articles published in health sciences research.

Methods

This bibliometric analysis study reviewed 10,596 articles published in PubMed in three 10-year intervals (1997, 2007, and 2017). The data were collected through Google sheet and were analyzed using SPSS software. This study described the trend and usage of currently available statistical tools and the different study designs that are associated with them.

Results

Of the statistical software mentioned in the retrieved articles, SPSS was the most common statistical tool used (52.1%) in the three-time periods followed by SAS (12.9%) and Stata (12.6%). WinBugs was the least used statistical software with only 40(0.6%) of the total articles. SPSS was mostly associated with observational (61.1%) and experimental (65.3%) study designs. On the other hand, Review Manager (43.7%) and Stata (38.3%) were the most statistical software associated with systematic reviews and meta-analyses.

Conclusion

In this study, SPSS was found to be the most widely used statistical software in the selected study periods. Observational studies were the most common health science research design. SPSS was associated with observational and experimental studies while Review Manager and Stata were mostly used for systematic reviews and meta-analysis.

## Introduction

With the evolution of open access in the publishing world, access to empirical research has never been more widespread than it is now. For most of the researchers, however, the key feature of their articles is the robustness and repeatability of their methods section particularly the design of the study and the type of statistical tests to employ. The emergency of statistical software has transformed the way scientists and researchers conducting their statistical analysis. Therefore, performing complex and at times erroneous statistical analysis manually has become thing of the past [1].

Statistical software has many useful applications for researchers in the healthcare sciences. Furthermore, the researchers conveniently read their data by representing their data as visual aids using charts and graphs [2]. It also helps the researchers to easily calculate their results using statistical tests by accounting for their variables either numerical, categorical, or both [2]. However, in the past few decades, statistical software usage went through different stages based on their development and applications [3]. Although some software are more dedicated to a specific field, the degree of usage of specific software may depend on the preference of the investigators or the type of study design that is selected in their research.

There are different types of statistical software and each of these is used for a different type of study. In a study of the popularity of statistical software in research, Muenchen R (2016) found that the Statistical Package for Social Sciences (SPSS) is by far the most popular statistical software used in epidemiological studies [3]. Another example of popular software that is used is SAS (Statistical Analysis Systems), which is statistical software that is considered to be flexible and has better graphical facilities compared to SPSS [4]. However, it can be difficult for novice users compared to SPSS due to its more advanced commands [4]. Another statistical software that is used in health sciences research is Stata. It allows users standard and non-standard methods of data analysis due to its ability to implement a powerful programming language in the analysis for a particular use. However, some of these features may be more difficult to use than other applications [5].

A study conducted in the United States has found that about 61% of original research articles in the Journal of Health Services Research have specified which statistical software they used for the data analysis [6]. Researchers also found that Stata and SAS were the predominant statistical software used in the reviewed articles. Another study about the use of statistical software and the various ways of measuring the popularity or their market share showed that SAS and SPSS were most popular in the business world. However, SAS was found to be the most popular in job openings followed by SPSS which had just less than double the Minitab, and R-project software had one-quarter of Minitab [7]. Another similar study was conducted in Pakistan which focused on the type of study design and the statistical software used in two local journals. The investigators found that SPSS was the most commonly used software while cross-sectional study design dominated the articles published [8].

Despite the development of the different statistical software and the speed with which these tools are produced, it may be very hard for the researchers to choose which statistical software they employ given the type of study design. This is complicated by the fact that all commercially available statistical tools strive to accommodate almost all features researchers need to analyze their data. Regardless of the type of study design, the availability of these statistical software, and the familiarity of the analysts in a particular software may greatly influence which statistical tool to use during the analysis. The type of statistical software and the choice of studies they are used for in health sciences is currently under-researched either because researchers have little knowledge about the applicability of specific statistical tools or the institutional decision on which software to use for analysis. To our knowledge, no study investigated the association between the use of statistical software and the chosen study design in different health sciences fields. Therefore, this study aimed to review the trend of the statistical software usage and their associated study designs among published health sciences articles in three time periods: 1997, 2007, and 2017.

## Materials and methods

This bibliometric web-based study covered 14 different statistical software. Because of health sciences being a vast and highly researched area, investigators have chosen to limit their search to one database, PubMed. This database comprises more than 30 million articles of health-related disciplines. The data collection was limited to a 10-year interval (1997, 2007, and 2017), and was accomplished from October 2018 to May 2019. The study employed a semi-structured review process to select the articles for the study. The main inclusion criterion was that the article selected used one of the following statistical software either mentioned in the abstract, methods, or in the full text if available: GraphPad, MedCalc, JMP, LISREL, WinBUGS, Review Manager, Microsoft Excel, Minitab, SAS, Epi Info, Stata, SPSS, Statistica, R Project. However, any article mentioned a similar name but did not mean the statistical software was excluded. The initial search generated 10,596 articles and the process used was as follows: All the 14 statistical software mentioned above were searched together using the Boolean operator “OR” as the connector word. Multiple names were included for the last software (R Project) because it had been mentioned in different articles with different names. Next, articles identified were filtered based on the specified periods which were 1997, 2007, and 2017. No additional filters or other restrictions such as the article language were applied.

For each selected article, the statistical software used and the study design employed were identified by reading the title and abstract. If none of the statistical software was found or the name was an acronym other than the software (e.g., SAS as SATB2-associated syndrome), then the article’s full text was examined. If the full text is not available, that article was excluded. Similarly, the study design was checked in the title and in the abstract and if not evident, the full text was reviewed if available. If two or more study designs were reported in the article, then the main study design was considered. Lastly, the PubMed identifier (PMID) number was added to avoid any errors or article duplications.

**Figure 1:**
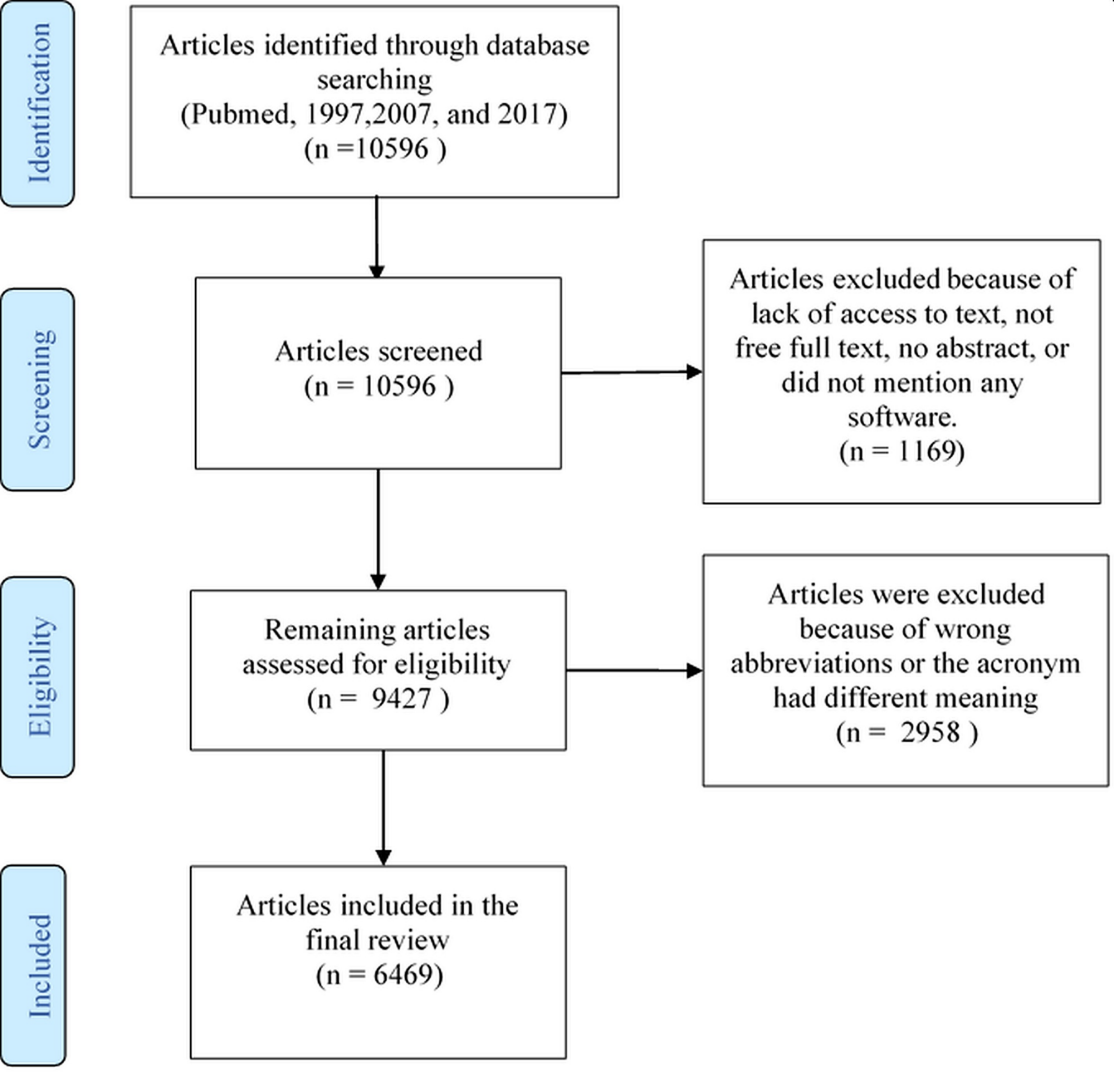
PRISMA flow diagram of the inclusion process of the articles.

Figure 1 represented the PRISMA flow diagram of the inclusion and exclusion process of the articles. The initial search for the three-time periods specified yielded 10,596 articles. Of those 1,169 articles were excluded because of lack of access to the abstract, the free full text or no software was mentioned. Furthermore, 2,958 articles were excluded because of wrong abbreviations or the acronym had a different meaning. Finally, 6,469 articles were included in the present study. The data were collected through a Google sheet and were analyzed using IBM SPSS Statistics for Windows, Version 24.0 (IBM Corp., Armonk, NY). Categorical data were presented as frequencies and percentages. Bar charts were used to display software usage across the study periods.

## Results

**Figure 2:**
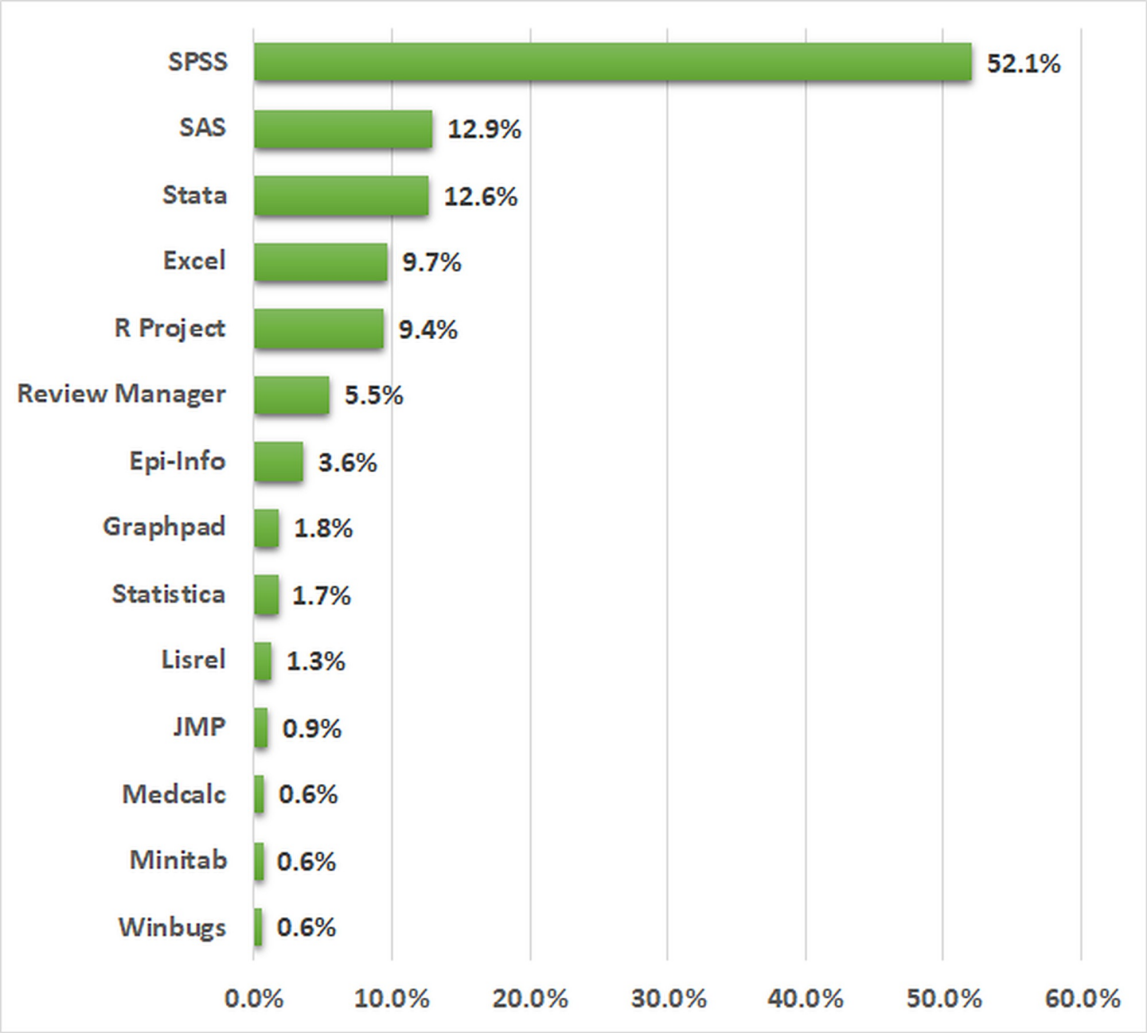
Percentages of the statistical software used in the reviewed articles. The total percentage was 113.3% since some articles used more than one software.

Of the 10,596 generated during the literature search, 6,469 articles that were published in the years 1997, 2007, and 2017 were included in the final review. The percentages of the statistical software used in these articles are shown in Figure 2. SPSS was the most commonly used statistical software for data analysis with 3,368 (52.1%) articles, followed by SAS 833 (12.9%), and Stata 815 (12.6%). WinBugs was the least used statistical software with only 40 (0.6%) of the total articles.

**Figure 3:**
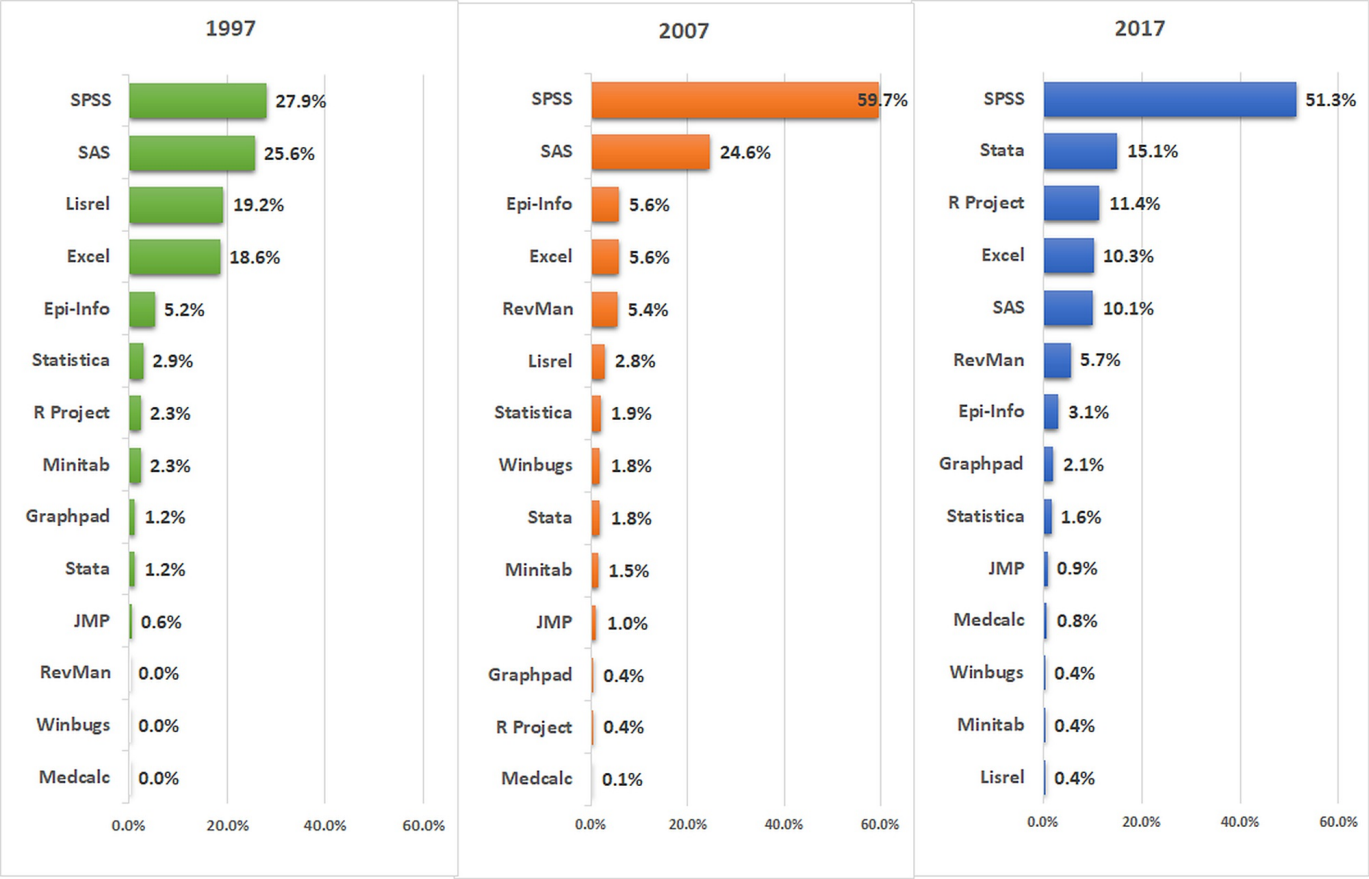
Statistical software used in medical research for each year of the study period (1997, 2007, and 2017).

As shown in Figure 3, SPSS was found to be the most commonly used statistical software throughout the study periods 1997 (27.9%), 2007 (59.7%), and 2017 (51.3%). SAS was second to SPSS in the first two periods while in 2017, its use has shifted down to fifth compared to Stata. Other software that have gained popularity included R-project and Review Manager. In the first time period (1997), the articles that used these tools were very few. However, in 2017, their use has shifted up to third (11.4%) and sixth (5.7%), respectively.

**Figure 4:**
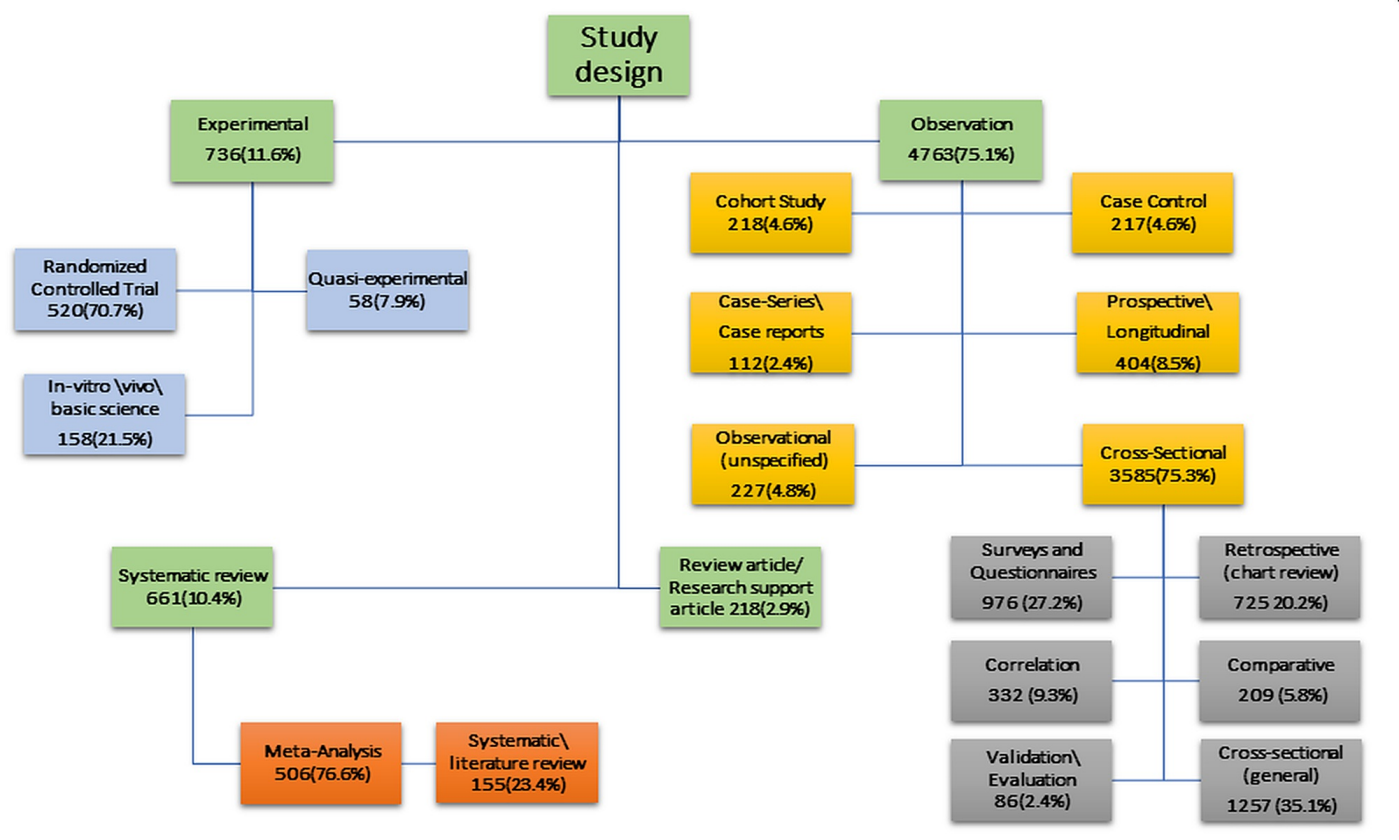
The study design used in the included articles.

Of the 6,469 reviewed articles, 6,342 (98%) had clearly mentioned study designs and the rest 127 (2%) were either not clear or not mentioned in the articles. The study designs were classified into four main types: observational 4,763 (75.1%), experimental 736 (11.6%), systematic review 661 (10.4%), and research support\review article 218 (2.9%) (Figure 4). Among the observational studies, cross-sectional was the most frequently used study design with 3,585 (75.3%). In experimental study designs, randomized controlled trials were the most used design with 520 (70.7%) in the reviewed articles. Around three-quarters of the systematic review articles, 506 (76.6%) also included meta-analyses.

Association between statistical software used and study design employed is shown in Table 1. The majority of the articles on systematic reviews‎\meta-analysis designs opted to use Review Manager (43.7%) followed by Stata (38.3%). Two-thirds of experimental studies used SPSS software for data analysis and only SAS software was the other major tool used in these studies. For the observational studies, again SPSS was the predominant statistical software used (61.1%) and the rest of the percentages were distributed among other statistical tools. Most review articles used R-project (60.2%) followed by SAS (27.7%) with only 6.6% of the review articles used SPSS.

**Table 1:** Association between statistical software used and study designs employed.

Software	Systematic review‎\meta-analysis (n = 661)	Experimental (n = 736)	Observation (n = 4,763)	Review articles/Research support (n = 218)
Epi-Info	0.0%	0.0%	5.9%	0.0%
Excel	5.3%	5.2%	8.6%	3.6%
Graphpad	0.4%	1.2%	0.7%	0.0%
JMP	0.1%	0.2%	0.6%	0.6%
Lisrel	0.0%	0.6%	1.3%	0.0%
Medcalc	0.6%	0.2%	0.5%	0.6%
Minitab	0.0%	0.4%	0.0%	0.0%
Review Manager	43.7%	0.0%	0.0%	0.0%
R-Project	6.9%	3.4%	4.0%	60.2%
SAS	0.9%	16.5%	7.5%	27.7%
SPSS	2.7%	65.3%	61.1%	6.6%
Stata	38.3%	5.2%	8.6%	0.6%
Statistica	0.0%	0.4%	1.0%	0.0%
Winbugs	1.1%	1.2%	0.2%	0.0%
Total	100.0%	100.0%	100.0%	100.0%

## Discussion

The relationship between the use of statistical software and the type of research designs in health sciences is not well understood. Therefore, the aim of this study was to describe the trends of the statistical software usage and their associated study designs among published health sciences articles in three time periods. While a five-year interval was possible, the number of articles required to be included would have been overwhelming. However, this study included articles published at a 10-year interval in 1997, 2007, and 2017. With the current search strategy, the amount of data collected exceeded 10,000 articles. One important issue during the data collection was the ambiguity in the abbreviation of names for the statistical software. For example, when typing the abbreviation SAS (Statistical Analysis System) on the PubMed search engine, the search results are sometimes mixed up with the abbreviation of sleep apnea scale or subarachnoid space, there was also a marked difference in software usage across all the years.

Overall, SPSS was found to be the most popular statistical software followed by SAS and Stata. When examined the use of the statistical software, SPSS was found to be the most popular tool in the chosen three time periods. The positions of the other statistical tools fluctuated in terms of their use in the health sciences. Regarding the associated study designs, observational studies and in particular cross-sectional were found to be the predominant when compared to other study designs. This study also found that SPSS was mostly used for observational and experimental studies while Review Manager and Stata were mostly associated with systematic reviews and meta-analysis.

This study included articles in all health sciences regardless of where they were published. However, unlike our study, some articles which reported the use of statistical software have limited their search to a specific region or local journals [6,8]. This study found that SPSS was the most used software worldwide. In contrast, a study conducted in the United States found that Stata was the most commonly used statistical software employed in health services followed by SAS [7]. Suggesting that there could be geographical variation in the use of statistical software. Another study conducted in Pakistan which included articles published in two local journals found that SPSS was the most commonly used statistical software [8].

Other reasons that may have caused the variation of the statistical software packages may include the availability of these tools in different parts of the world and the preferences of the researchers. In the US study, for example, close to 50% of US-based researchers used Stata while the percentage of non-US articles that used Stata was only 15% [7].

For the study design, the current study found that around three-quarters of observational studies were cross-sectional. Our finding agreed with a study conducted in Saudi Arabia which reported almost a similar percentage [9]. However, the Pakistani study found half the percentage of both studies [8]. Regarding the other study designs, only 10.4% of the articles were systematic reviews or meta-analyses in this study. This lower percentage found in this study agrees with a study in the United States that investigated the relationship between the type of study design and the chances of citation in the first two years. They reported that only 4% of the 624 articles were meta-analyses or systematic reviews [10].

Limitations

Because of logistical and personnel issues, the current study only used the PubMed database. Lack of access to the full text of the retrieved titles caused a number of articles to be excluded. This may have introduced bias in reporting the type of statistical software or the chosen study design. This study depended on the reported study designs and did not verify their accuracy, as it was not the main aim of the study.

## Conclusions

The purpose of this study was to inform researchers about the usage of the different statistical software packages and their associated study designs in health sciences research. In this study, SPSS was found to be the most widely used statistical software throughout the whole study period. The observational studies were the dominating health science research design with cross-sectional studies being the most common study design. SPSS was associated with observational and experimental studies while Review Manager and Stata were mostly used for systematic reviews and meta-analysis. As this the first wide-ranging study about the statistical software use and the associated study designs, we envisage that it will be of benefit to researchers to choose the most probable statistical software regarding their chosen study design.
